# Comprehensive landscape and future perspectives of exosomal circular RNAs in cardiovascular disease

**DOI:** 10.3389/fphar.2026.1860253

**Published:** 2026-08-06

**Authors:** Yuan Zheng, Qianhui You, Yi Shu, Yurui Yuan, Zhuoyue Zhang, Ting Chen, Baonian Liu

**Affiliations:** 1 Department of Anatomy, School of Integrative Medicine, Shanghai University of Traditional Chinese Medicine, Shanghai, China; 2 Shenzhen Bao’an Authentic TCM Therapy Hospital, Guangzhou University of Chinese Medicine, Shenzhen, China; 3 Chinese PLA General Hospital, Medical School of Chinese PLA, Beijing, China; 4 Oncology Department, Suzhou TCM Hospital Affiliated to Nanjing University of Chinese Medicine, Suzhou, Jiangsu, China

**Keywords:** biomarker, cardiovascular disease, circular RNA, exosomal circRNA, exosome, therapeutic target

## Abstract

Cardiovascular diseases continue to be a predominant cause of global mortality, driven by pathological molecular transport networks. Exosomes, which encapsulate diverse molecular cargo reflective of their cellular origins and associated pathological states, serve as valuable resources for identifying diagnostic and prognostic biomarkers. An increasing number of studies conducted in recent years have shown that exosomal circular RNA (circRNA) is important for a variety of cardiovascular diseases (CVDs), such as coronary heart disease, atherosclerosis, senescence, myocardial infarction, and myocardial damage. Recent research has highlighted the critical functions of circRNAs, which operate through various mechanisms, including the regulation of gene expression by serving as microRNA (miRNA) sponges, operating a dual synergistic mechanism that simultaneously promotes cytokinesis and cell cycle restoration, which enhances myocardial repair, and directly combines RNA-binding proteins and ligases to promote the recognition and degradation of proteins. Exploring the mechanisms underlying CVDs from the perspective of exosomal circRNA is of great significance. It has far-reaching value for the prevention, early diagnosis, and effective treatment of cardiovascular disorders. This review summarizes the latest investigation on the hyperlink between CVDs and exosomal circRNA.

## 1 Introduction

Human lifespan is greatly impacted by cardiovascular disease (CVD), which also raises the chance of disability and early death (Berry et al., 2012). The results highlight the importance of prevention, early diagnosis, and effective treatment of CVDs. Even with progress in drug and intervention treatments, the ongoing increase in CVD morbidity and mortality highlights significant shortcomings in addressing the factors driving CVD advancement (Romero-Cabrera et al., 2022; Zhang M. et al., 2024). Exosomal circular RNAs (circRNAs) in the etiology and management of cardiovascular disorders have been extensively investigated over the past decade (Granger and Emambokus, 2015).

Exosomes are membrane-bound vesicles secreted by nearly all living cells, which serve as pivotal mediators of intercellular communication (Liang et al., 2021; Sun et al., 2025; Wang et al., 2025). Exosomes, usually 40–160 nm in diameter, play a regulatory role in cardiac apoptosis, cardiomyocyte hypertrophy, blood pressure regulation, angiogenesis, and other processes (Kalluri and LeBleu, 2020; Hade et al., 2021). They are indeed highly diverse in their molecular composition, encapsulating a wide array of biomolecules such as lipids, proteins, DNA, and various RNA species, including circRNA, microRNA (miRNA), and long non-coding RNA (lncRNA) (Pegtel and Gould, 2019; Chen et al., 2025). Furthermore, exosomes isolated from patients with CVD are enriched in pro-inflammatory factors, indicating their potential processes in the initiation and progression of CVD (Wang et al., 2021).

Circular RNA (circRNA) represents a distinct class of non-coding RNA (ncRNA) molecules with a continuous loop. Accumulating evidence indicates that circRNAs have the ability to function as miRNA decoys, thereby sequestering them and modulating their activity (Li Z. et al., 2021; Zhu et al., 2022; Nie et al., 2023). Furthermore, binding to RNA-binding proteins (RBPs) is a common mechanism by which circRNAs exert their functions. Some circRNAs contain m6A modification or internal ribosome entry sites (IRES), which can be translated into proteins or peptides under specific circumstances, producing short peptides with biological functions, thereby affecting gene expression and participating in biological systems, such as cell differentiation, proliferation, and apoptosis (Qin et al., 2021; Zhang Y. et al., 2023; Habara, 2025). A subset of these circRNAs is implicated in pathological vascular processes, including the dysfunction of endothelial cells, the enhanced proliferation and migration of vascular smooth muscle cells (VSMCs), and the promotion of inflammatory responses within the vasculature (Wang et al., 2022; Xu et al., 2022; Singh et al., 2023). These observations imply that circRNAs have tremendous promise for the prevention and treatment of cardiovascular complications (Kristensen et al., 2019). In addition, a body of evidence suggests that circRNAs are tightly associated with many important CVDs, such as atherosclerosis (AS), myocardial hypertrophy, myocardial infarction, pulmonary hypertension, and atrial fibrillation (Mei and Chen, 2022). CircRNAs are detected across multiple blood compartments with distinct characteristics. Whole blood contains the highest absolute abundance of circRNAs, primarily derived from blood cells such as platelets and leukocytes, but this compartment is susceptible to cellular contamination and hemolysis artifacts. In contrast, plasma and serum harbor cell-free circRNAs, predominantly encapsulated within extracellular vesicles such as exosomes, with a smaller fraction bound to proteins.

Meanwhile, there is a distinction between exosomal circRNAs and circulating circRNAs derived from plasma or whole blood that is largely methodological rather than strictly biological. Techniques routinely used to isolate total RNA from plasma, serum, or whole blood yield a heterogeneous mixture that inherently includes exosomal circRNAs, as the majority of stable, RNase-resistant circRNAs in the circulation are packaged within extracellular vesicles. It is undeniable that a significant number of studies have examined the effects of circRNA in plasma and whole blood on CVDs. For instance, the expression levels of plasma circRNAs can represent promising therapeutic candidates for CVDs, such as circDICAR, whose expression is significantly lower in cardiomyocyte pyroptosis patients compared to healthy controls (Yuan et al., 2023). Furthermore, electrochemical approaches have been developed for whole blood circRNA detection, such as using a collaborative CRISPR-Cas system as a proof-of-concept platform for acute myocardial infarction (Sha et al., 2024).

Among the various types of circRNAs in the circulatory system, exosomal circRNAs exhibit the highest circRNA-to-linear RNA ratio, superior stability conferred by the lipid bilayer, and traceability to their parental cells, making them ideal candidates for studying intercellular communication and biomarker discovery (Chen C. et al., 2022). While total plasma circRNA profiling offers higher absolute yields and greater practicality for large-scale screening, exosomal circRNA analysis provides enhanced specificity and mechanistic insight. Nonetheless, there is a lack of up-to-date summary treatises on articles examining the link between exosomal circRNAs and CVD. For these reasons, we selected exosomal circRNAs as the subject of a dedicated review.

**FIGURE 1:**
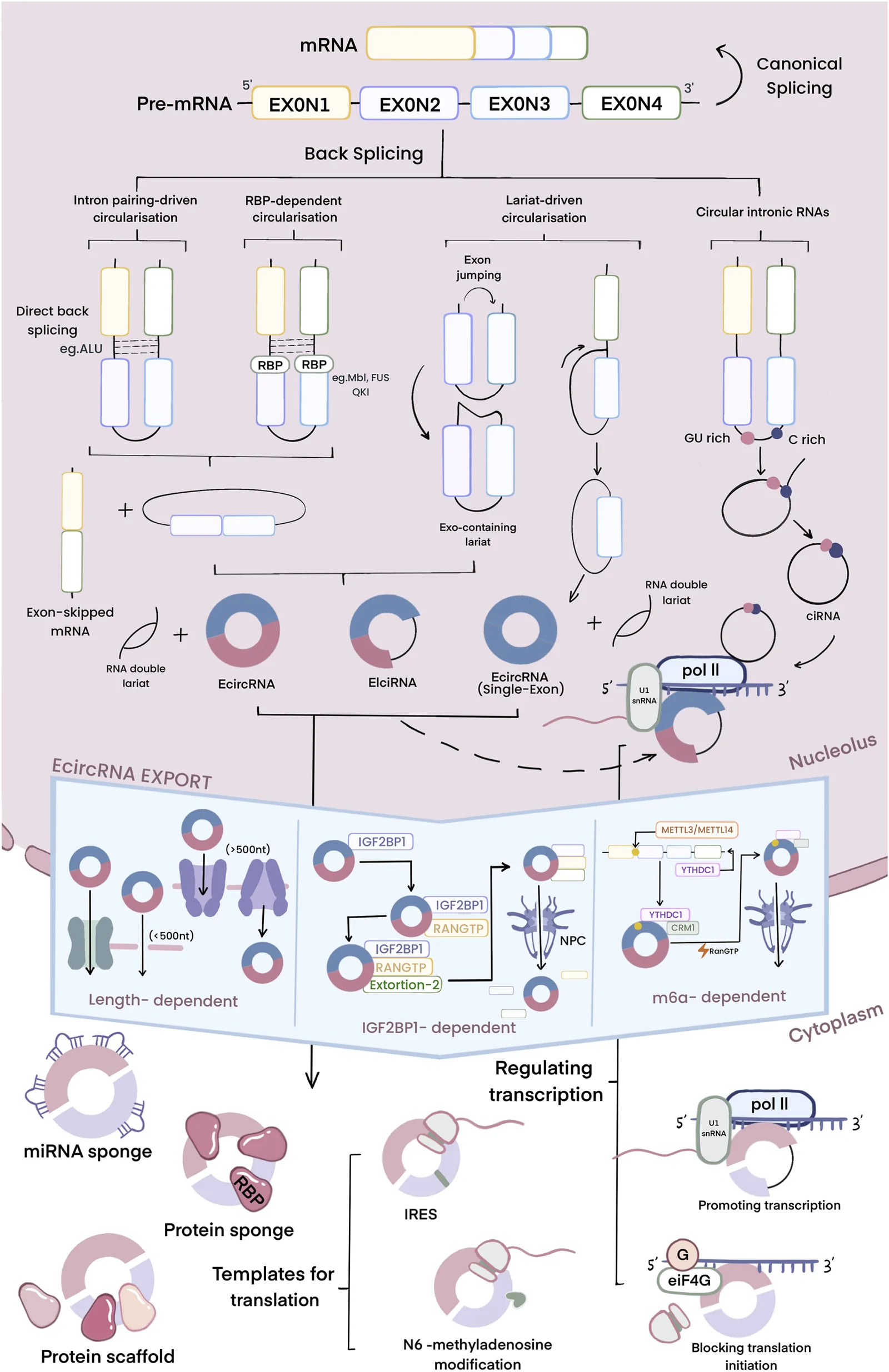
The biogenesis, subclasses, and functions of circRNAs. CircRNAs constitute a class of ncRNAs generated by a unique back-splicing mechanism. Their formation mainly occurs through these pathways: the lariat-driven model, intron pairing-driven circularization, RBP-dependent circularization, and circular intronic RNA pathways. Depending on their genomic composition, circRNAs are classified into several subtypes, including exonic circRNAs (EcircRNAs), ciRNAs, exon-intron circRNAs (EIciRNAs), and ciRNAs. These molecules exert diverse biological roles by acting as miRNA sponges, modulating the splicing of precursor mRNAs, and serving as scaffolds for protein interactions.

**FIGURE 2:**
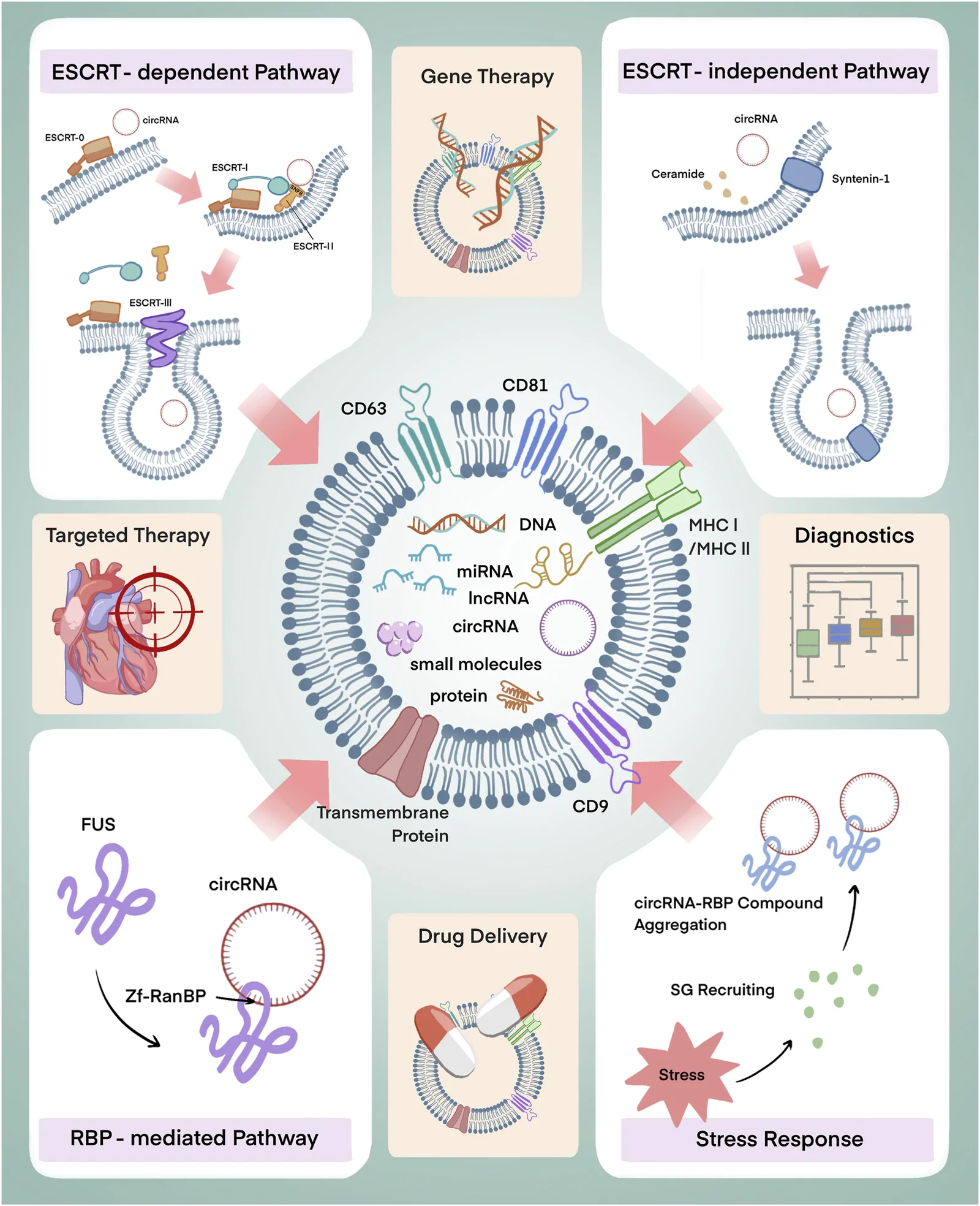
Bioactive molecules on exosomes, the mechanisms of circRNAs’ entry into exosomes, and the use strategies of exosomes. Various bioactive molecules on exosomes can be used as biomarkers for disease. Exosomes consist of diverse crucial biomolecules, such as the four transmembrane protein family (CD9, CD63, CD81), growth factors (TGFB 1, bFGF), major histocompatibility complexes (MHC I/MHC II), adhesion proteins, heat shock proteins, enzymes, lipids, nucleic acids, and a small portion of genetic material, of which CD9, CD63, CD81 and HSP70Alix (Qiu et al., 2024). The mechanisms by which circRNAs enter exosomes include the ESCRT-dependent pathway, the ESCRT-independent pathway, the RBP-mediated pathway, and the stress response.

In this review, we detail the circRNA lifecycle, encompassing biogenesis, biosynthesis, functions, and degradation from the beginning (Figure 1). Secondly, we explain how circRNAs enter and become incorporated into exosomes, and what functions they perform (Figure 2). Next, we investigated the physiological function, biological structure, and influence of exosomal and circRNAs on the etiology of CVDs. In particular, the article emphasizes the mechanisms by which exosomal circRNAs exert their influence across different CVDs and their significance for disease diagnosis and treatment, and highlights their broad application prospects and scientific value. This study will demonstrate that the research on exosomal circRNAs can help elucidate the pathological mechanisms of CVDs.

## 2 Overview of exosomes

### 2.1 Basic characteristics of exosomes

Exosomes are vesicular structures formed by membranes and membrane-bound molecules (Salunkhe et al., 2020). They are usually 40–160 nm in diameter. Exosomes generally form their final mature form by inward growth of the bounding membrane. They are eventually discharged outside the cell via plasma membrane fusion and are one typical form of extracellular vesicles (Batrakova and Kim, 2015).

Exosomes contain a variety of biomolecules, including specific protein macromolecules and various nucleic acid molecules. These biomolecules can often be secreted outside the vesicle in some way to perform specific functions, such as regulating cellular activity or acting as messengers for intercellular communication (Boriachek et al., 2018).

The morphology of exosomes has been diversified in different kinds of cells, and even in the same kind of cells, a high degree of diversity can be maintained (Li W. et al., 2021). To confirm this phenomenon, the visualization and characterization of exosomes can be achieved through multiple electron microscopy-based methods. These techniques encompass transmission electron microscopy, cryo-electron microscopy, and scanning electron microscopy (Kishore et al., 2016; Gao et al., 2019).

### 2.2 Physiological functions of exosomes

Functioning as intercellular messengers, exosomes carry diverse molecular cargo, including RNAs and proteins (Hegde et al., 2023; Pan et al., 2025). Their native lipid bilayer structure, derived from the cell membrane, facilitates efficient uptake through fusion with target cells, enabling the delivery of their contents (Weaver et al., 2022). They primarily facilitate intercellular communication and transport molecular cargo to recipient cells. Additionally, exosomes contribute to tissue and organ regeneration. They also have an impact on cell migration, death, differentiation, and proliferation (Zhong et al., 2021). At the same time, exosomes have contributed to macroscopic life activities. For example, emerging evidence indicates that exosomes released by hypothalamic neural stem cells contribute significantly to delaying the aging process (Cui et al., 2024). In addition to aging, emerging research highlights the significant impact of exosomes on reproductive processes and embryonic development. They participate in critical reproductive stages and are pivotal mediators of maternal-fetal immunological crosstalk throughout gestation (He et al., 2018; Wang C. et al., 2024; Xie et al., 2025).

Meanwhile, exosomes are a particular form of vesicular structure, which can help communicate between different cells and even tissues through, for example, membrane fusion (Shi et al., 2024). Recent studies have demonstrated that the transfer of information through exosome transport is an important way of intercellular communication, with a status equivalent to that of direct intercellular contact and the transfer of information through soluble molecules. For instance, exosome-mediated communication plays a crucial role within the nervous system. These vesicles facilitate bidirectional signaling between various neural cell types, including sensory neurons, motor neurons, and glial cells (He et al., 2018).

Moreover, due to the powerful function of exosome transportation, some types of exosomes can receive miRNA or lncRNA for intercellular transfer, which ultimately leads to changes in biological phenotypes, such as the development of drug resistance (He et al., 2018).

### 2.3 Roles of exosomes in pathological processes

Exosomes are implicated in a wide range of pathological processes, including CVDs, viral pathogenesis, reproductive health, central nervous system disorders, immune regulation, and oncogenesis. Exosomes’ intrinsic ability to alter complex intracellular signaling cascades supports their potential therapeutic use for several illnesses, especially cancer and neurological diseases (Kalluri and LeBleu, 2020; Hu et al., 2024).

#### 2.3.1 Cardiovascular diseases

Exosomes play a significant role in the cardiovascular system, especially in cardiomyocyte hypertrophy, myocardial fibrosis, blood pressure regulation, and anti-apoptotic effects. (Virani et al., 2020). Cells including cardiac fibroblasts, vascular, cardiac progenitors, cardiomyocytes, stem cells, and endothelial cells release exosomes (Hade et al., 2021).

For instance, exosomes are involved in AS by promoting inflammation and plaque formation. Studies indicate that exosomes derived from bone marrow mesenchymal stem cells (MSCs) are capable of maintaining myocardial architecture. Exosomes promote coagulation by exposing anionic phospholipids, especially phosphatidylserine, and by exposing tissue factor, the trigger of the clotting system, showing that exosomes have a dual role in hemostasis with procoagulant and fibrinolytic properties (Coumans et al., 2017). At the same time, MSC-exosomes confer cardioprotection by inhibiting cardiomyocyte hypertrophy, reducing apoptosis, suggesting a potential therapeutic avenue for heart failure (Pan et al., 2023). Collectively, numerous studies have indicated that endogenous exosomes can improve cardiac function following cardiac stimulation. This evidence supports novel treatment approaches for CVDs, although their translation requires further clinical investigation.

#### 2.3.2 Other diseases

Tumor-derived exosomes contribute to cancer progression by enhancing cell proliferation, stimulating angiogenesis, and mediating resistance to therapeutic agents (Cai et al., 2021). Exosomes mediate intercellular transfer of oncogenic molecules, thereby remodeling the tumor microenvironment as well as inducing immunosuppression. A representative mechanism involves circNRIP1 sequestering miR-149–5p. This sponge effect leads to the upregulation of AKT1 expression, ultimately promoting gastric cancer progression (Guo et al., 2021).

Studies also indicate that exosomes can deliver viral proteins, miRNAs, and viral RNA components, thereby promoting the spread of viral infection between tissue cells (Wu et al., 2022). Exosomes are critically involved in mediating the neuroinflammatory process (Joseph et al., 2022). By presenting autoantigens in an immunogenic manner, exosomes are capable of directly stimulating autoreactive T cells in the peripheral immune system (Almohaimeed et al., 2025). These vesicles can regulate immune responses by affecting the activation of T cells and strengthening regulatory pathways to promote immune tolerance (Lin and Aware, 2026).

Despite their role in pathology, owing to their innate role in intercellular communication, prolonged circulation half-life, and high biocompatibility, exosomes are promising candidates for drug delivery systems. They are well-suited for transporting diverse cargo, including chemical drugs, proteins, nucleic acids, and gene therapy agents (Liu and Su, 2019).

## 3 Overview of circRNA

### 3.1 Features of circRNA

CircRNAs refer to a distinct category of ncRNAs, defined by their covalently closed, continuous loop structure formed via a unique back-splicing mechanism (Xu et al., 2023), which endows them with distinct biological properties compared to conventional linear RNAs. The circular conformation of circRNAs endows them with exceptional stability, rendering them resistant to degradation by RNA exonucleases. Consequently, they exhibit a substantially extended half-life relative to mRNAs across most species (Liu et al., 2025). CircRNA resists Ribonuclease R because of its circular structure, which gives it stability within the cell and prevents destruction (Wang T. et al., 2024). In the meantime, it has been shown that circRNA is broadly dispersed, created endogenously in cells, specialized to certain cell and tissue types, and specific to particular developmental stages (Liao et al., 2020). Furthermore, because of their intrinsic stability, evolutionary conservation, and abundant content in body fluids, circRNAs are considered promising biomarkers for various pathological conditions (Zhang et al., 2018).

CircRNAs can be divided into four categories, as listed: exonic circRNAs, intronic circRNAs, exon-intronic circRNAs, and intergenic circRNAs (Chen L. D. et al., 2022; Shang et al., 2022), of which exonic circRNAs account for up to 80% of all circRNAs and are mainly formed through exon post-flip junctions. Exonic circRNAs can be further classified based on the number of exon fragments they contain. Intronic circRNAs, which are produced through the circularization of intron sequences via a distinct splicing mechanism, are predominantly localized within the cell nucleus. Formed by intronic reverse transcription, thus mainly distributed in the nucleus, which is more favorable to the regulation of expression and transcription of specific genes; other types of circRNAs are mainly distributed in the cytoplasm after formation, competing with other RNAs for adsorption of proteins and performing their respective functions; and intergenic circRNAs are circRNAs derived from intergenic sequences or other unexpressed genomic sequences. Notably, the category of intron-derived circRNAs encompasses not only circular intronic RNAs (ciRNAs) but also other forms such as excised group I and group II introns, spliced tRNA introns, and various intronic sequence variants (Hu et al., 2022; Liu et al., 2024d).

CircRNA biogenesis constitutes a pivotal non-canonical splicing pathway. The integrated Figure 1 delineates a coordinated molecular cascade encompassing circRNA formation, processing, and function (Figure 1). The foundational pathway of canonical linear splicing serves as a comparative reference, wherein precursor mRNA undergoes conventional spliceosomal processing to yield mature linear transcripts. In contrast, circRNA genesis is predominantly driven by back-splicing—a sophisticated molecular rearrangement wherein the 3′splice donor of a downstream exon forms a covalent ligation with the 5′splice acceptor of an upstream exon, resulting in a closed circular transcript. This circularization event is orchestrated through four principal molecular mechanisms, each exhibiting distinct regulatory characteristics. Intron-pairing-driven circularization leverages complementary sequences, notably ALU repeats, within flanking introns to facilitate splice site approximation, which is also known as direct back splicing. Alternatively, RBP-dependent circularization involves specific trans-acting factors—including Muscleblind, Fused in Sarcoma, and Quaking proteins—that recognize cognate intronic motifs and establish molecular bridges between distal splice sites. A lariat-driven pathway exploits exon-jumping intermediates, where excised lariat structures undergo debranching and subsequent backsplicing to generate circular isoforms. Furthermore, a specialized subclass emerges through circular intronic RNA formation, wherein introns containing conserved GU-rich 5′elements and C-rich branchpoint motifs evade debranching enzymes to form stable circular structures (Figure 1).

Following biogenesis, these circular transcripts undergo stringent subcellular sorting—while exon-intron circRNAs demonstrate nuclear retention with preferential nucleolar localization, exonic circRNAs are predominantly exported to the cytoplasmic compartment via specific transport machinery. While intron-retaining circRNAs generally remain nuclear, most circRNAs are exon-derived and must be actively exported to the cytoplasm to execute functions such as miRNA sponging and translation. Their nuclear export employs dedicated, active transport pathways.

A primary, conserved mechanism for ecircRNA export is the insulin-like growth factor 2 (IGF2BP1)-dependent pathway. Recent findings reveal that the RBP IGF2BP1 binds specifically to sequence or structural motifs within many circRNAs. In the nucleus, Ran-GTP enhances IGF2BP1 association with circRNA and simultaneously binds to the export receptor Exportin-2. This assembly forms a circRNA–IGF2BP1–Exportin-2–Ran-GTP quaternary complex, which is translocated through the nuclear pore. In the cytoplasm, hydrolysis of Ran-GTP to Ran-GTP triggers disassembly, releasing the circRNA. This pathway underscores an IGF2BP1-dependent, active transport process analogous to protein export (Figure 1) (Ngo et al., 2024; You et al., 2026).

CircRNA export is also regulated in a length-dependent manner by DEAD-box RNA helicases. Short circRNAs (<∼500 nucleotides) predominantly require URH49. While long circRNAs (>∼500 nucleotides) rely on UAP56 (Wan and Hopper, 2018). Specific RNA modifications and RBPs can license or enhance nuclear egress for subsets of circRNAs, such as N^6^-methyladenosine (m^6^A) Modification. The presence of m^6^A on certain circRNAs is recognized by the nuclear reader YTHDC1, which promotes their export, paralleling a mechanism observed for some m^6^A-modified mRNAs (Figure 1) (You et al., 2026).

### 3.2 Functions of circRNA

CircRNAs harbor multiple miRNA response elements, capable of specifically binding to miRNAs, thus enabling circRNAs to function as efficient molecular sponges, acting as the most critical function of circRNAs. For instance, extracellular circRNAs are capable of facilitating critical intercellular crosstalk, such as between cardiomyocytes and endothelial cells, and between M2 macrophages and cardiac fibroblasts. These communication pathways are implicated in the pathophysiology of ischemic myocardial injury and subsequent cardiac remodeling (Li B. et al., 2022). By sequestering specific miRNAs, they prevent the miRNAs from binding to and repressing their target mRNAs, thereby influencing various biological functions as well as regulating downstream gene expression (Liu et al., 2026). Some of the circRNAs are highly active against RBPs, which can influence their function or localization, acting as protein-binding sites (Pala and Yilmaz, 2025) (Figure 1).

Similarly, circRNAs can serve as molecular scaffolds and sponges that selectively bind RBPs, thereby influencing the subcellular localization and stability of host gene-encoded proteins. This binding activity can promote the sequestration, compartmentalization, or transport of specific proteins, leading to a net reduction in the functional availability of target proteins within critical cellular regions (Huang et al., 2022). Likewise, although traditionally classified as ncRNAs, a portion of circRNAs was found to have protein-coding potential. The translation of these circRNAs can yield functional peptides or proteins, and the dysregulation of such products is implicated in oncogenesis and other pathologies (Hamdy et al., 2025). When it comes to templates for translation, engineered RNA circles or endogenous circRNAs with an embedded IRES are able to allow cap-independent translation, and circRNAs engaged with m6A modifications allow cap-independent translation (Liu and Chen, 2022) (Figure 1). Owing to their structural stability and dysregulated expression in pathological states, circRNAs are recognized as emerging and valuable candidates for both diagnostic biomarkers and therapeutic targets (Dostalova Merkerova et al., 2020).

### 3.3 Roles of circRNA in pathological processes

CircRNAs are highly expressed in various biological compartments, including peripheral blood, specific tissues and organs like the brain and human umbilical vein endothelium (Xiao et al., 2021). They exert multifaceted regulatory functions in various pathological processes through several distinct mechanisms, such as miRNA or RBP decoys, sequestering mRNAs, regulating transcription, and, in some cases, directly encoding functional polypeptides (Zhang et al., 2018). Notably, circRNAs are critical regulators in heart development and disease. A notable example is MICRA (Myocardial Infarction-associated CircRNA), which was investigated for its prognostic value in patients following acute myocardial infarction. In a study of 472 myocardial infarction patients, Salgado-Somoza et al. measured MICRA levels in whole blood samples collected at the time of reperfusion and assessed left ventricular ejection fraction (EF) at 4 months post-myocardial infarction. Using the three-tier classification system recommended by the European Society of Cardiology, the authors found that lower MICRA expression was significantly associated with a higher risk of developing left ventricular dysfunction. Multivariable ordinal regression analysis revealed that MICRA independently classified patients into EF groups and provided incremental predictive value beyond established clinical markers, including NT-proBNP and creatine phosphokinase. Bootstrap internal validation further confirmed that MICRA was among the top predictors selected in the majority of models. This study highlights the potential of blood cell-derived circRNAs as stable, accessible biomarkers for refining post-MI risk stratification and guiding personalized therapeutic strategies (Salgado-Somoza et al., 2017).

For example, CiRS-7, in numerous cases, functions as a potent molecular sponge for miR-7, thereby modulating its activity. This interaction provided the initial demonstration that circRNAs can play the role of competing endogenous RNA (ceRNAs) to sequester miRNAs (Greene et al., 2017).

Furthermore, the role of circRNAs as regulators in CVD, primarily through sequestering miRNAs and RBPs, has recently attracted considerable research attention. Their detection in bodily fluids, with expression patterns altered by disease or development, strongly supports their utility as circulating biomarkers for CVD. Advancing methodologies to map the spatiotemporal expression of circRNAs across diverse cardiovascular cell types will be crucial for elucidating their precise regulatory functions under both biological and pathological circumstances. (Kishore et al., 2020). Additionally, circRNAs can regulate the interactions of pro-angiogenic factors and glioma growth factors to induce angiogenesis (Sun et al., 2021).

### 3.4 Exosomal circRNA

#### 3.4.1 Features of exosomal circRNA

Exosomal circRNAs exhibit functional versatility. Beyond their roles in binding miRNAs or proteins, they are packaged into multivesicular endosomes (MVEs) alongside other abundant molecular freight, such as nucleic acids, lipids, and proteins (Manini et al., 2019).

#### 3.4.2 Generation of exosomal circRNA

Numerous nucleic acid molecules, such as circRNA, which is produced inside the cell and enters the exosome by a particular channel to carry out its role, are among the inclusions found in exosomes (Aswad et al., 2014). Many potential pathways have been investigated in current research, but the precise mechanism of selection for circRNA entrance into exosomes is still unknown. The incorporation of circRNAs into exosomes occurs through multiple mechanisms, including pathways dependent on endosomal sorting complexes required for transport (ESCRT), as well as ESCRT-independent pathways, RBP-mediated sorting, and cellular stress responses (Henne et al., 2011; Wang J. et al., 2024; Chen et al., 2026; Du et al., 2026) (Figure 2). According to several researchers, miRNAs are essential for the circRNAs’ targeted entry into exosomes and subsequent destruction. It has been shown that the competitive endogenous sponge circCDR1as in exosomes was significantly reduced upon simulating mechanical stimulation of cells with miR-7 (Hu et al., 2022).

The bioactive molecules carried by exosomes, the processes governing circRNA loading into these vesicles, and the therapeutic strategies involving exosomes are key research areas. Notably, specific bioactive molecules on exosomes act as valuable biomarkers for diseases (Yin et al., 2025). CircRNAs enter exosomes through four fundamental processes, including the ESCRT-dependent pathway, ESCRT-independent pathway, RBP-mediated pathway, and stress response. Within the ESCRT-dependent pathway, specific sorting of circRNAs into exosomes is mediated through interaction with sucrose non-fermenting 8 (SNF8), a component of the ESCRT-II complex, facilitated by unique RNA motifs. Alternatively, exosome formation can proceed through an ESCRT-independent pathway, relying on proteins such as CD63 and lipids like ceramides (Zubkova et al., 2024). In the non-ESCRT-dependent pathway, ceramide and syntenin-1 can drive the budding of the endosomal membrane from ILV, by which circRNA enters, Rab27a/b, and Rab35. The trafficking of multivesicular bodies (MVBs) to the plasma membrane for exosome release is regulated, and the fusion of MVBs with the plasma membrane is mediated by the soluble N-ethylmaleimide-sensitive factor attachment protein receptor (SNARE) complex. In the RBP-mediated pathway, such as the zinc finger RBP domain of fused in sarcoma (FUS), which recognizes the junction sequence of circRNA (Wharton et al., 2025), FUS enters the cytoplasm and co-localizes with stress granules (SGs) underoxia, carrying circRNA into exosomes. Similarly, the packaging of circRNAs into exosomes is also modulated in response to external stimuli or cellular stress conditions. SG recruits and then circRNA-RBP compound aggregates. When the stimulation is over, they will be transported into exosomes for secretion.

#### 3.4.3 Function of exosomal circRNA

Exo-circRNAs are released from donor cells into the extracellular space and subsequently internalized by recipient cells (Wang et al., 2020). Once internalized, they can influence the recipient cell’s behavior by regulating protein function, gene expression, or signaling pathways (Luo and Gui, 2020). This makes them crucial for maintaining tissue homeostasis and coordinating responses in multicellular organisms.

Exosomal circRNAs commonly function as molecular decoys, either sequestering proteins or stabilizing and sponging miRNAs (Mafi et al., 2024). The abundance of exosomal circRNAs is modulated by intracellular fluctuations in their cognate miRNA levels. This packaged molecular information is then delivered to recipient cells, where it participates in modulating physiological processes. The above pathways mainly mediate the expression and degradation levels of circRNAs in exosomes by regulating miRNAs. In addition, some researchers believe that RBP is also a key substance in regulating the entry of circRNA into exosomes. In this pathway, RBP regulates the selective entry of circRNAs by binding to specific sequences (Hu et al., 2022). The sorting of miRNAs has been explicitly shown to be facilitated by the participation of certain RBPs, such as heterogeneous nuclear ribonucleoproteins and RNA-induced silencing complex catalytic component 2, which identify and bind to certain sequence patterns in miRNAs (Guo et al., 2024). It has also been found that RBP is enriched in the exosomes of DKs-8 cells, and RBP promotes the entry of circFAT1 into exosomes in a binding manner (Hu et al., 2022).

Exosome-encapsulated circRNAs serve multiple roles. They function by interacting with miRNAs and proteins, and are co-packaged into MVEs alongside a diverse molecular payload that includes other nucleic acids, lipids, and proteins (Wang et al., 2019). Following their secretion from parental cells into bodily fluids, exosomes enable the systemic circulation of circRNAs, allowing them to exert their biological effects. Furthermore, they also take part in regulating fundamental cellular processes, including development, proliferation, and invasion (Gao et al., 2021). Additionally, circRNAs are under investigation for their utility in monitoring disease progression and recurrence, tracking chemotherapy response, and aiding in the development of vaccines and therapeutics, with applications in personalized medicine. Concurrently, multiple clinical trials and research studies are actively assessing the feasibility of employing circRNAs as biomarkers (Latifi-Pakdehi et al., 2024). For example, in many CVDs, the use of exosomal circRNAs can help to detect the disease at the very beginning, which can contribute to the discovery and treatment of diseases that do not have obvious symptoms in the early stages.

In CVD, exosomal circRNAs play diverse roles through various molecular mechanisms. These include serving as miRNA sponges, acting as protein scaffolds or recruiters, contributing to polypeptide translation, and interacting with RNA. Furthermore, recent experimental studies have consistently demonstrated that exosomal circRNA can serve as a biomarker for the clinical diagnosis of CVDs, warranting further research and development (Table 1).

**TABLE 1:** Exosomal circRNAs exert multiple molecular functions in cardiovascular diseases.

Circular RNA	Injury model	Expression	Target signaling pathway	Biological roles	References
miRNA sponge					
CircELP3 (circ_0001785)	Atherosclerosis	Upregulated	MiR-513a-5p/TGFBR3	Reduce endothelial cell injury and delay atherogenesis	Tong et al. (2023a)
Circ_0006896	Atherosclerosis	Upregulated	Circ_0006896-miR-1264-DNMT1 axis	Promote destabilization and rupture of carotid artery plaques	Wen et al., (2021)
CircCERS5 (circ_0026218)	Endothelial dysfunction	Upregulated	MiR-188–3p/TLR4/NF-κB pathway	Promote endothelial dysfunction	Liu J. et al., (2024)
Circ_0004104	Endothelial dysfunction	Upregulated	MiR-942–5p/ROCK2 axis	Attenuate endothelial dysfunction	Zhang Y. et al., (2022)
CircIFNGR2 (circ_0002113)	Myocardial injury	Upregulated	Circ_0002113/miR-188–3p/RUNX1 axis	Exacerbate myocardial cell apoptosis and myocardial infarction	Tian et al., (2022)
Circ_0018553	Cardiac hypertrophy	Downregulated	MiR-4731/SIRT2 signaling pathway	Promote cardiomyocyte proliferation, inhibits apoptosis, and induces hypertrophy	Zuo et al. (2023)
CircASXL1	Coronary heart disease	Downregulated	circASXL1/miR-1/CDK6/Rb1/cell-cycle	Promote the re-entry of cardiomyocytes into the cell cycle	Wang Y. et al. (2024)
Circ_0086296	Atherosclerosis	Downregulated	Circ_0086296/miR-576–3p/IFIT1/STAT1 axis	Accelerate the formation of atherosclerotic plaques	Zhang M. et al. (2022)
Circ_Stt3b	Coronary heart disease	Upregulated	Circ_Stt3b/miR-15a-5p/GPX4 signaling	Reduce myocardial damage following a heart attack	Liu et al. (2024b)
CircCCDC66 (circ_0001312)	DOX-induced cardiotoxicity	Upregulated	MiR-409–3p/HMGB1 axis	Promote cardiomyocyte apoptosis, inflammation, and oxidative stress	Hu et al. (2022)
Circ_100696	Atherosclerosis	Upregulated	Circ_100696/miR-503–5p/PAPPA axis	Accelerate the progression of atherosclerosis	Liu et al. (2023a)
CircChordc1 (circ_0001747)	Myocardial injury	Downregulated	MiR-199b-3p/MCL1 axis	Promote cardiomyocyte proliferation and inhibits apoptosis and inflammation	Zhou et al. (2022)
CircSLC8A1	Myocardial injury	Upregulated	CircSLC8A1/miR-214–5p/TEAD1 axis	Exacerbate hypoxia-induced myocardial injury	Lan et al. (2022)
CircZNF292	Myocardial injury	Downregulated	CircZNF292/miR-146a-5p axis	Protect cardiomyocytes from IH-induced apoptotic damage	Xie et al. (2024)
CircHIPK3	Heart failure	Downregulated	MiR-33a-5p/IRS1 axis	Promote H_2_O_2_-induced proliferation of AC16 cardiomyocytes and inhibits apoptosis	Fan et al. (2023)
CircRTN4	Myocardial injury	Downregulated	CircRTN4/miR-497–5p/MG53↑pathway	Mitigate sepsis-induced myocardial injury	Li J. et al. (2022)
Hsa_circ_0012627	Endothelial dysfunction	Downregulated	CeRNA network	Contributing to PM2.5-induced vascular endothelial dysfunction	Liu Q. et al. (2024)
Hsa_circ_0074698	Dilated cardiomyopathy	Upregulated	Extracellular ligand–receptor sequestration pathway	Cardioprotective effects in DCM patients with CHF.	Xu et al. (2024)
CircELP3 (circ_0001785)	Coronary heart disease	Downregulated	Circ_0001785/miR-513a-5p/TGFBR3 axis	Delay atherogenesis	Tong et al. (2023)
Protein scaffold					
CircHIPK3	Cardiac senescence	Downregulated	E3/β-TrCP/HuR/p21 axis	Inhibits myocardial cell aging and maintains cardiac function	Ding et al. (2022)
Protein recruiter					
CircCDR1	Atherosclerosis	Upregulated	IgE–FcεR1–Exosome–CDR1as–FUS–phos-p65	Promote atherosclerotic lesion	Yang et al. (2024)
Peptide translation					
CircMYO9A (circ_0036176)	Cardiac fibrosis	Upregulated	Myo9a-208/cyclin/Rb/axis	Inhibits the proliferation of cardiac fibroblasts	Guo J. et al. (2022)
RNA interaction					
CircASXL1	Coronary heart disease	Downregulated	circASXL1/Ncl/Ribo-bio axis	Promote cytokinesis	Wang Y. et al. (2024)

## 4 Exosomal circRNAs in cardiovascular diseases

**FIGURE 3:**
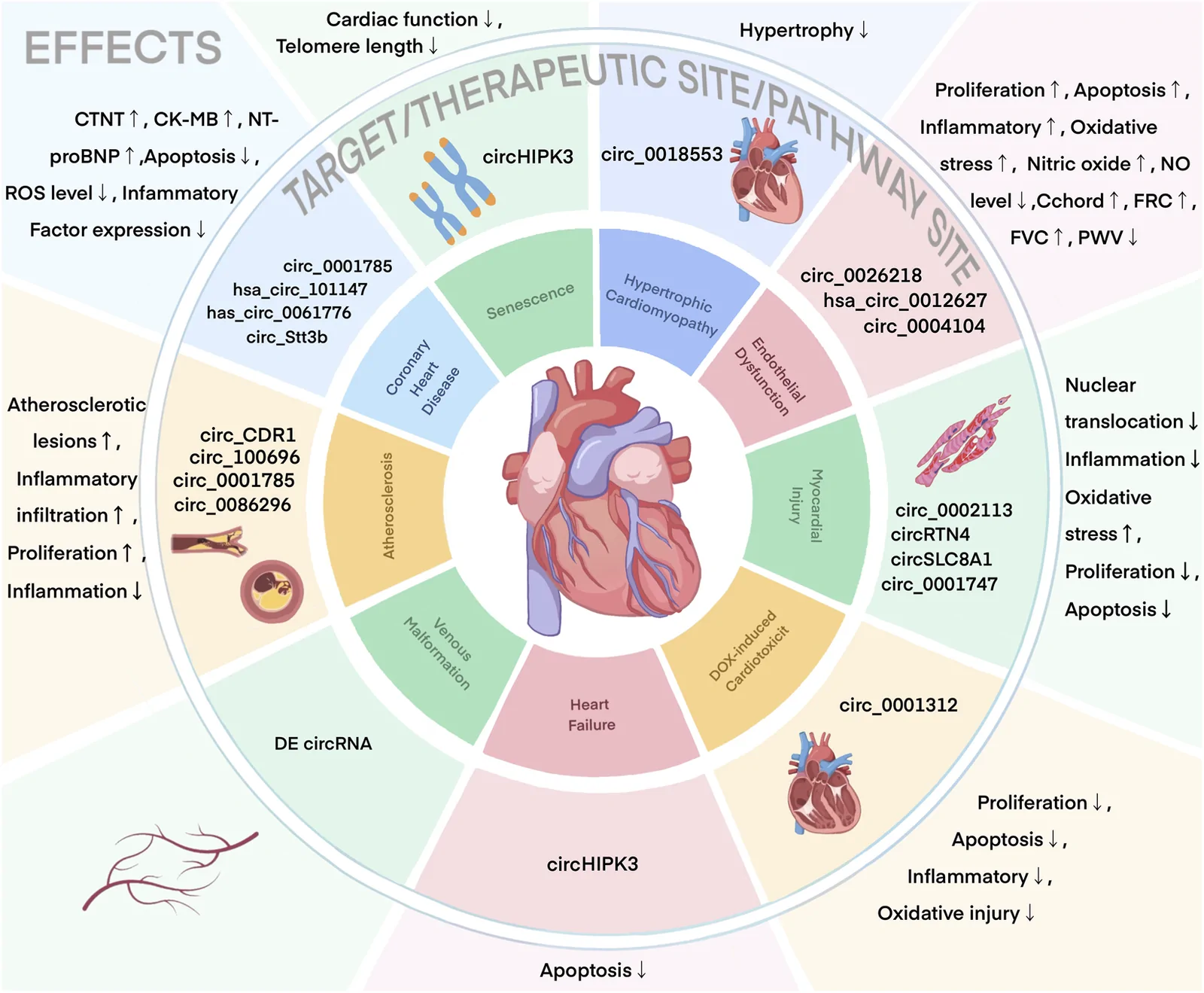
Diagram of the functions of exosomal circRNAs in various aspects of CVD. The functions of exosomal circRNAs in CVD include CHD, AS, heart failure, and so on, making exosomal circRNAs potential biomarkers and therapeutic targets.

There has been growing attention to those studies of exosomal circRNA in CVD lately. A systematic literature search was performed using the PubMed database to identify all published research articles investigating exosomal circRNAs in the context of CVD from 2021 to 2025. These articles cover multiple subdivided diseases or action pathways within the scope of CVDs, which vividly show the role of exosomal circRNA in cardiovascular system diseases and provide ideas for the design as well as application of therapeutic targets (Figure 3; Table 2).

**TABLE 2:** Exosomal circular RNAs in cardiovascular diseases.

Injury model	Exosomes	Intervention methods	Circular RNA	Effects	Targets or pathways	References
Coronary heart disease	Circulating exosomes	None	Hsa_circ_0001558↑etc.	CTNT↑, CK-MB↑, NT-proBNP↑	None	Liu X. et al. (2024)
Coronary heart disease	Exosomes from patients with CHD	None	CircELP3↓ (circ_0001785, circ_0000973, circ_0001741, circ_0003922.)	None	None	Tong et al. (2023b)
Coronary heart disease	Exosomes from OSA with AMI patients	None	Hsa_circ_101147↑, Hsa_circ_101561↑etc.	None	MiR-29a-3p/hsa_circ_104642 pathways↑	Huang. et al. (2023)
Coronary heart disease	ADSC exosomes	*In vitro*: hypoxic-pretreated ADSC exosomes *In vivo*: MI mouse model	Circ-Stt3b↑	Apoptosis↓, ROS level↓, Inflammatory factor expression↓	Circ-Stt3b/miR-15a-5p/GPX4 signaling activation↑	Liu J. et al. (2024)
Coronary heart disease	Plasma exosomes	None	Exo-hsa_circ_0075269↑, Exo-hsa_circ_0000284↑	None	None	Liu X. et al. (2023)
Coronary heart disease	Exosomes from eukaryotic cells of STEMI patients	None	Circ_0020887↑, Circ_0009590↑	None	None	Wang et al. (2023)
Coronary heart disease	Blood exosomes	None	Hsa_circ_0001360↑, Hsa_circ_0000038↑	None	NET↑/NOD-↑	Zhang W. et al. (2024)
Coronary heart disease	Plasma exosomes	None	Has_circ_0061776↑	None	None	He et al. (2022)
Coronary heart disease	UMSC-Exos	*In vivo*: Rb1 knockout mice	CircASXL1↓	Proliferation↓, cell-cycle reentry↑, Cytokinesis↑	CircASXL1/CDK6/Rb1/cell-cycle reentry↑	Wang et al. Y. (2024)
Myocardial injury	Exosomes derived from circRNA_0002113	*In vitro*: anoxia-reoxygenation (A/R) model cells	CircIFNGR2↑ (circ_0002113)	Nuclear translocation↓, Apoptosis↓	Circ_0002113/miR-188–3p↓/RUNX1↑ axis	Tian et al. (2022)
Myocardial injury	Mesenchymal stem cells-derived exosomes	*In vivo*: cecal ligation and puncture (CLP) rats *In vitro*: LPS-treated cardiomyocytes	CircRTN4↓	Oxidative stress↓ Inflammation↓ Cardiac dysfunction↓	CircRTN4/miR-497–5p/MG53↑ pathway	Li B. et al.(2022)
Myocardial injury	Hypoxic cardiomyocyte exosomes	None	CircSLC8A1↑	Cell viability↓, Apoptosis↑, Inflammatory cytokines↑, oxidative stress↑	CircSLC8A1/miR-214–5p/TEAD1↑ axis	Lan et al. (2022)
Myocardial injury	ADSC exosomes	*In vivo*: mice hypoxia/reoxygenation (H/R) injury model	CircChordc1↓ (circ_0001747)	Cell viability↑, Proliferation↓, Apoptosis↓, Inflammation↓	MiR-199b-3p↓/MCL1↑ axis	Zhou et al. (2022)
Myocardial injury	AC16 exosomes	*In vitro*: qRT-PCR	CircZNF292↓	Apoptosis↑	CircZNF292↓/miR-146a-5p axis↑	Xie et al. (2024)
Atherosclerosis	Mast cell exosomes	*In vivo*: atherosclerosis mice, FcεR1 knock-out mice	CircCDR1↑	Atherosclerotic lesions↑, Inflammatory infiltration↑	IgE-FcεR1↑	Yang et al. (2024)
Atherosclerosis	Macrophage exosomes	*In vitro*: cell model of AS	Circ_100696↑	Proliferation↑, cell cycle↑, cell migration↑	Circ_100696/miR-503–5p/PAPPA axis↑	Liu J. et al. (2023)
Atherosclerosis	Serum exosomes	*In vitro*: human umbilical vein endothelial cells *In vivo*: lentivirus	CircELP3↑ (circ_0001785)	Inflammation↓, Apoptosis↓, endothelial cell injury↓	MiR-513a-5p↓/TGFBR3↓	Tong et al. (2023)
Atherosclerosis	Exosomes from ECs	*In vitro*: oxidized low-density lipoprotein treated HUVECs *In vivo*: atherosclerotic mice model	Circ_0086296↓	Atherosclerotic lesion formation↓	Circ_0086296/miR-576–3p/IFIT1 axis	Zhang et al.M. (2022)
Atherosclerosis	Serum exosomes	*In vitro*: HUVECs incubated with serum-Exos	Circ_0006896	Carotid plaque destabilization↑	Circ_0006896-miR-1264-DNMT1 axis↑	Wen et al. (2021)
Atherosclerosis	Human blood exosomes	None	Hsa_circ_0005699	None	None	Gu et al. (2021)
Endothelial dysfunction	Exosomes from HUVECs	*In vitro*: cell model of AS	CircCERS5↑ (circ_0026218)	Proliferation↑, Apoptosis↑, Inflammatory↑, oxidative stress↑, Nitric oxide↑, NO level↓	MiR-188–3p↑/TLR4/NF-κB pathway↓	Liu et al. (2024)
Endothelial dysfunction	BEAS-2B cell exosomes	*In vivo*: the ambient-PM2.5-exposed mice	Hsa_circ_0012627↓etc.	Cchord↑, FRC↑, FVC↑, PWV↓	Migration and EMT in BEAS-2B cells↑	Liu Q. et al. (2024)
Endothelial dysfunction	HUVEC exosomes	*In vitro*: ox-LDL-exposed HUVECs	Circ_0004104↑	Proliferation↓, Apoptosis↑, angiogenesis ability↓, protein levels↓, Inflammation↑	MiR-942–5p↓/ROCK2 axis↓	Zhang Y. et al. (2022)
Heart failure	AC16 cell exosomes	*In vitro*: hydrogen peroxide exposure	CircHIPK3↑	Apoptosis↓	MiR-33a-5p/IRS1 axis↑	Fan et al. (2023)
Cardiac fibrosis	AC16 cardiomyocyte exosomes	*In vitro*: cell model of cardiac fibrosis	Circ_0036176↑	Proliferation↓	Cyclin/Rb signal↓	Guo et al. (2022)
Cardiac hypertrophy	EPC-derived exosomes	*In vitro*: Ang II-induced cardiac hypertrophy	Circ_0018553↓	Cardiac hypertrophy↓	MiR-4731↓/SIRT2 signaling pathway↓	Zuo et al. (2023)
Cardiac senescence	UMSC-Exos	*In vivo*: cardiomyocyte-specific senescent animal model	CircHIPK3↓	Cardiac function↓, telomere length↓	Binding of E3 ubiquitin ligase β-TrCP and HuR↑, p21 activity↓	Ding et al. (2022)
DOX-induced cardiotoxicity	Cardiomyocyte exosomes	None	CircCCDC66↑ (circ_0001312)	Proliferation↓, Apoptosis↓, Inflammatory↓, oxidative injury↓	MiR-409–3p/HMGB1↑ axis↓	Hu et al. (2022)
Dilated cardiomyopathy	Plasma exosomes	None	Hsa_circ_0074698↑etc.	None	None	Xu et al. (2024)
Limb vascular disease	Serum exosomes	None	Hsa_circ_0001842↑	None	None	Liu Q. et al (2023)
Venous malformation	Serum exosomes	None	CircASAP1↑	None	Thyroid hormone signaling pathway↑	Zhang L. et al. (2023)

### 4.1 Coronary heart disease

Coronary heart disease (CHD) is predominantly caused by the development of AS in the coronary arteries. This pathological process induces myocardial ischemia and hypoxia. Globally, CHD represents the main cause of incidence and mortality (Li M. et al., 2022). The intricate mechanics of CHD have been better interpreted thanks to current research. But there are still a lot of residual hazards.

Exosomal circRNAs have been increasingly recognized as key molecular players in the pathogenesis of CVDs, with a substantial body of research dedicated to elucidating their roles. According to the research of Tong et al., the screening of exosomal circRNAs from CHD patients revealed 85 differentially expressed species, with 81 downregulated and 4 upregulated. Utilizing predictive data for circRNAs, miRNAs, and their target mRNAs, a ceRNA network was constructed. Serum exosomes were isolated from patients with CHD and healthy controls and co-cultured with human umbilical vein endothelial cells *in vitro*. Through dual-luciferase reporter assays, circ_0001785 (circELP3) was identified as a ceRNA involved in coronary artery disease. Functional studies employing overexpression lentiviral transfection confirmed the protective role of circ_0001785 against endothelial cell injury. Furthermore, *in vivo* experiments demonstrated that tail-vein injection of circ_0001785-overexpressing lentivirus exerted therapeutic effects in a mouse model of AS. Validation via quantitative real-time PCR (qRT-PCR) identified four specific circRNAs—circ_0001785, circ_0001741, circ_0000973, and circ_0003922—as promising predictive biomarkers for CHD. Most importantly, researchers have found that overexpression of circ_0001785 could reduce endothelial cell injury through the pathway of miR-513a-5p/TGFBR3. These outcomes offer valuable insights for the investigation into the pathogenesis of CHD and acute coronary syndrome (Tong et al., 2023b).

Beyond qualitative investigations into molecular pathways, quantitative circRNAomics—the systematic profiling of circRNA expression—represents a promising frontier for deciphering disease processes. Liu and colleagues carried out high-throughput sequencing on the plasma exosomal RNA of 15 chronic coronary syndrome (CCS) patients and 15 non-cardiac chest pain patients to screen for differentially expressed circRNAs, which reported a distinct expression profile of circRNAs in plasma exosomes taken from patients with CCS compared to controls. Their study identified exosomal hsa_circ_0075269 and hsa_circ_0000284 as potential novel diagnostic biomarkers for CCS. (Liu X. et al., 2023). Similarly, according to Zhang et al., the regulatory network, obtained from the exoRBase database, including lncRNAs, circRNAs, miRNAs, and mRNAs, may provide mechanistic insights into CHD. The combination of hsa_circ_0001360 and hsa_circ_0000038 shows promise as a significant biological marker (Zhang et al., 2024b).

All these studies clearly demonstrate the role of exosomal circRNA as a biomarker in CHD, playing a key role in disease pathway networks. Six circRNAs—circ_0001785, circ_0001741, circ_0000973, circ_0003922, hsa_circ_0001360, and hsa_circ_0000038 —were validated as promising predictive biomarkers of CHD. Importantly, circ_0001785 was found to mitigate endothelial cell injury. Meanwhile, exosomal hsa_circ_0075269 and hsa_circ_0000284 were identified as potential diagnostic biomarkers for CCS, providing valuable insights for future research and diagnostic applications in terms of detection methods and data characteristics of CHD. However, these studies suffer from small sample sizes and lack statistical significance. Further research is urgently needed to explore these topics in greater depth.

Among CHD, myocardial infarction represents a major contributor to mortality and disability among patients with CVD globally. Myocardial infarction is the cell death of cardiomyocytes caused by a severe and persistent ischemic state due to the result of an imbalance in perfusion between supply and demand in the heart.

In the research of Huang et al., high-throughput sequencing was performed to analyze the serum exosomal circRNA profiles in three groups: healthy subjects, patients with obstructive sleep apnea (OSA) but without AMI, and OSA patients with AMI. Bioinformatics analyses were subsequently employed to identify potential core circRNAs, and functional analyses were conducted to investigate their associated biological roles. The qRT-PCR analysis confirmed the differential expression of two circRNAs (hsa_circ_101147 and hsa_circ_101561) between healthy subjects and patients with OSA without AMI, as well as four circRNAs (hsa_circ_101328, hsa_circ_104172, hsa_circ_104640, and hsa_circ_104642) between healthy subjects and OSA patients with AMI. Furthermore, the researchers verified that miR-29a-3p directly targets hsa_circ_104642. The study identified multiple dysregulated exosomal circRNAs from patients with OSA and concomitant AMI, which have the potential to be further developed as a basis for clinical diagnosis. (Huang et al., 2023).

Similarly, in the area of clinical research, plasma expression profiles of exosomal circRNAs in patients with AMI, stable coronary heart atherosclerotic disease, and healthy controls obtained from a GEO expression dataset, the research by He and colleagues delineated the functional roles of exo-circRNAs in AMI, offering deeper insights into the potential pathogenic mechanisms involving an exosomal circRNA-mediated ceRNA network in AMI (He et al., 2022).

In the context of myocardial infarction and the subsequent cardiac repair process, circASXL1 delivered by umbilical cord mesenchymal stem cell exosome (UMSC-Exo) was found to precisely regulate the initiation (DNA synthesis) and completion (cytokinesis) of the cell cycle, respectively, through two parallel signaling pathways. Experimental verification shows that knockdown of circASXL1 in cardiomyocytes will reduce CDK6 expression, while knocking down Rb1 can effectively promote cardiomyocyte DNA synthesis and entry into the G2/M phase. Studies have found that simply knocking out Rb1 (activating pathway one) can only briefly improve heart function in the early stages after myocardial infarction, but it cannot achieve long-term repair. Because it only promotes DNA replication, without addressing the problem of cytoplasmic division. However, the exosome of circASXL1 activates both of the above pathways, both starting the cell cycle and providing the ribosomes needed to complete division, thus achieving lasting repair. Exosomes derived from UMSC-Exo promote myocardial repair following infarction by inducing cardiomyocyte cell-cycle reentry and cytokinesis, a process dependent on circASXL1. This technique offers a potentially effective treatment approach to improve heart regeneration. (Wang Y. et al., 2024).

Another finding with potential clinical diagnostic value is that, in the findings of Liu et al., high-throughput sequencing was performed to profile differentially expressed circRNAs in circulating exosomes of AMI patients. For validation, real-time polymerase chain reaction was used to assess five circRNAs that showed significant upregulation. Variations between individuals experiencing non-cardiac chest discomfort and those suffering from acute myocardial infarction (AMI) were marked. Notably, hsa_circ_0001558 shows potential as a diagnostic marker for AMI (Liu et al., 2024e).

According to Liu et al.'s work, to assess oxidative stress and cell death under hypoxic conditions, reactive oxygen species levels and apoptosis in HL-1 cardiomyocytes were analyzed via immunofluorescence and flow cytometry. In addition, a mouse model of myocardial infarction was established, and the therapeutic efficacy of exosomes was evaluated through immunohistochemistry, immunofluorescence, and enzyme-linked immunosorbent assay. The researchers found that overexpression of circ_Stt3b enhanced the therapeutic efficacy of adipose-derived stem cell exosomes (ADSC-Exos) in alleviating post-MI cardiac injury. The findings indicate that exosomes from hypoxia-pretreated ADSCs attenuate cardiac damage after MI by activating the circ_Stt3b/miR-15a-5p/GPX4 signaling axis and suppressing ferroptosis (Liu et al., 2024a). Moreover, using reverse transcription and qRT-PCR, elevated levels of plasma exosomal circ_0020887 and exo_circ_0009590 were found to serve as potential indicators for diagnosing ST-elevation myocardial infarction and predicting short-term adverse cardiovascular events in these patients in Wang’s research (Wang et al., 2023).

Currently, the research on circRNA in CHD includes not only extensive research on exosomal circRNA as biomarkers, but also the identification of various mechanisms of exosomal circRNA in CHD, some of which have been shown to offer unique advantages in the treatment of CHD. Several studies have identified distinct exosomal circRNA signatures in CHD. In AMI, circRNAs such as hsa_circ_0001558, circ_0020887, and circ_0009590 show diagnostic potential. In CCS, hsa_circ_0075269 and hsa_circ_0000284 emerge as candidate biomarkers. For OSA patients with AMI, a panel including hsa_circ_104642 is dysregulated. Functional studies reveal that specific circRNAs, like circ_0001785, protect against endothelial injury via the miR-513a-5p/TGFBR3 axis, while circ_Stt3b in stem cell exosomes mitigates post-infarct damage by regulating the miR-15a-5p/GPX4 pathway to inhibit ferroptosis. Moreover, circASXL1 delivered by UMSC-Exo promotes durable cardiac repair by coordinately regulating cardiomyocyte cell-cycle re-entry and cytokinesis. In-depth research into potential pathogenic mechanisms and circRNA expression profiles will inevitably become a trend, and more research is required to determine its function in clinical therapy. Despite promising findings, current evidence is often constrained by limited sample sizes. Further validation in larger cohorts and deeper mechanistic investigations are needed to translate these exosomal circRNAs into reliable clinical tools for diagnosis, prognosis, and therapy in CVD (Figure 3; Table 2).

### 4.2 Atherosclerosis

Atherosclerosis (AS), known as a chronic disease, is a vital trigger of numerous CVD episodes, including CHD and heart failure. AS occurs mainly in the lining of many medium and large arteries, especially at the bifurcations of blood vessels. As a major chronic disease, AS has resulted in a global public health burden.

The role of exosomal circRNA as a biomarker for disease detection has been the most extensively studied and validated. Wen et al. investigated the differential expression profiles of serum exosomal circRNAs between patients with stable plaque AS (SA) and unstable/vulnerable plaque AS (UA). Through circRNA microarray analysis and RT-qPCR validation, they identified that circ_0006896 was significantly upregulated in serum exosomes from UA patients compared to those from SA patients. Notably, circ_0006896 expression positively correlated with triglyceride, low-density lipoprotein cholesterol (LDL-C), and C-reactive protein (CRP) levels, and negatively correlated with albumin levels in UA patients, suggesting its association with pro-inflammatory and pro-atherogenic states. Mechanistically, circ_0006896 was found to act as a competitive endogenous RNA (ceRNA) by sponging miR-1264, thereby relieving the repression of its target gene DNMT1. Upregulation of DNMT1 led to suppressed expression of SOCS3 via promoter hypermethylation, subsequently activating STAT3 phosphorylation. Functional assays demonstrated that UA serum exosomes promoted human umbilical vein endothelial cell (HUVEC) proliferation and migration through this circ_0006896–miR-1264–DNMT1 axis, implicating exosomal circRNAs in endothelial dysfunction and plaque destabilization. This study highlights the potential of exosomal circ_0006896 as both a biomarker for vulnerable plaques and a therapeutic target in AS (Wen et al., 2021). Similarly, Gu et al. identified hsa_circ_0005699 and the seven identified exosomal genes that could help to elucidate the pathogenesis and progression of atherosclerosis by examining a microarray dataset (Gu et al., 2021).

There are also studies on atherosclerosis that focus on the molecular functions of exosomal circRNA. In Tong and his colleagues’ study, circRNAs played a “sequester miRNA” role. They explored the role and specific mechanism of exosomal-derived circRNA circ_0001785 in the onset and progression of AS. Circ_0001785 works as a “molecular sponge” to adsorb miR-513a-5p, releasing the inhibition on the target gene transforming growth factor beta receptor 3 (TGFBR3), forming a competitive endogenous RNA network of circ_0001785/miR-53a-5p/TGFBR3, and then playing a defensive role in endothelial cells, ultimately delaying the progression of AS. The known target gene of miR-513a-5p is TGFBR3. Under normal circumstances, miR-513–5p will bind to the mRNA of TGFBR3, reducing the expression of TGFBR3 protein. When circ_001785 is overexpressed, it adsorbs a large amount of miR-513a-5p, thus relieving the inhibition of miR-513a-5p on TGFBR3. TGFBR3 expression is therefore upregulated. Their findings indicate that exosome-derived circ_0001785 is able to mitigate endothelial cell injury and attenuate atherogenesis. This mechanism presents a potential exosomal therapeutic method for AS (Tong et al., 2023a). This research revealed the typical mode of action of exosomal circRNAs in AS—exerting as a sponge for miRNAs.

Additionally, the study conducted by Yang et al. found that utilizing mouse models fed a standard diet and FcεR1-knockout mice, research demonstrated that exosomes released from IgE-stimulated mast cells exacerbate AS. This effect is mediated by exosomal circRNA CDR1as, which induces endothelial dysfunction. A cohort study revealed that patients with atherosclerosis who also had allergies exhibited higher plasma levels of CDR1as compared to both non-allergic atherosclerosis patients and healthy controls. (Yang et al., 2024). These results provide fresh perspectives on the connection between AS and exosomal circRNAs. highlighting potential diagnostic and therapeutic targets.

Furthermore, Liu et al. demonstrated the effect of circ_ 100,696 (circBase ID: hsa_circ_0008896) in AS. Knockdown of this molecule abolished the stimulatory effects of macrophage-derived exosomes (OM-Exo) on the proliferation and migration of VSMCs. Circ_100696 acts as a molecular sponge for miR-503–5p, which in turn directly binds to and regulates pregnancy-associated plasma protein A (PAPPA). VSMC migration and proliferation were enhanced by Circ_100696 overexpression, these effects were counteracted by either upregulating miR-503–5p or silencing PAPPA. Also, circ_100696 upregulated PAPPA expression by sponging miR-503–5p. OM-Exo was shown to stimulate VSMC growth and motility, thus contributing to the onset of AS (Liu J. et al., 2023). Similarly, exosomal circRNA has also been shown to promote the development of AS through other pathways. Inhibition of the circ_0086296/miR-576–3p/IFIT1 axis might prevent the development of atherosclerotic lesions both *in vivo* and *in vitro*. Therefore, the circ_0086296/miR-576–3p/IFIT1/STAT1 feedback loop plays a role in the progression of AS and contributes to the elevated levels of circ_0086296 observed in the exosomes of serum from patients with AS. (Zhang M. et al., 2022) (Figure 3; Table 2).

Emerging research demonstrates that exosomal circRNAs critically regulate AS progression primarily by acting as ceRNAs or “molecular sponges” that sequester miRNAs, thereby modulating the expression of target genes involved in vascular pathology. One well-characterized pathway involves circ_0001785, which functions as a decoy for miR-513a-5p, leading to the derepression of its target TGFBR3. This circ_0001785/miR-513a-5p/TGFBR3 axis exerts a protective effect by mitigating endothelial cell injury and attenuating atherogenesis. Conversely, other exosomal circRNAs promote AS progression. For instance, CDR1as in exosomes derived from IgE-stimulated mast cells induces endothelial dysfunction and exacerbates plaque formation. Similarly, macrophage-derived exosomal circ_100696 sponges miR-503–5p, leading to upregulation of PAPPA, which in turn stimulates vascular smooth muscle cell proliferation and migration—key events in AS development. Another feedback loop involves the circ_0086296/miR-576–3p/IFIT1/STAT1 axis, wherein elevated exosomal circ_0086296 levels promote atherosclerotic lesion formation both *in vitro* and *in vivo*. Collectively, these studies highlight the dual role of exosomal circRNAs in AS—either protective or pathogenic—through specific ceRNA networks. Undoubtedly, the aforementioned exosomal circRNA mechanism will further contribute to the comprehension of the pathogenesis of AS and will hopefully increase the likelihood of successful treatment.

### 4.3 Senescence

One important pathogenic factor in the development of CVDs is cellular senescence. This state is defined by markers such as elevated p21 expression and telomere attrition. The progressive accumulation of pathological senescent cells contributes to tissue necrosis and the pathogenesis of various cardiovascular disorders. Currently, effective therapeutic strategies to modulate the senescence process remain limited.

Ding et al. established an animal model of cardiomyocyte-specific senescence and revealed the underlying mechanisms through experiments. By administering circHIPK3 (hsa_circ_0000284 and mmu_circ_0001052), UMSC-Exos have anti-aging and cardioprotective benefits. The specific mechanism is that circHIPK3 facilitates the ubiquitination and degradation of human antigen R (HuR) by bringing the ubiquitin E3 ligase beta-transducin repeat-containing protein (β-TrCP) and the RBP HuR close to each other as molecular “scaffolds”, ultimately slowing down the decrease in the level of cardiomyocyte function, offering a promising therapeutic strategy for aging-related cardiac dysfunction (Ding et al., 2022).

This finding suggests that exosomes mediate cardioprotection by delivering circHIPK3. This represents a novel direction for diagnosing and treating cardiac dysfunction associated with myocardial senescence, while also providing a theoretical foundation for developing new therapeutic approaches and pharmaceutical agents. (Figure 3; Table 2).

### 4.4 Heart failure

Heart failure is a group of circulation problems brought on by compromised heart diastolic and/or systolic function. The most general cause of edema and dyspnea is heart failure. In heart failure, the heart’s impaired pumping capacity fails to meet the body’s circulatory demands unless ventricular filling pressures are elevated. This results in venous congestion and inadequate arterial perfusion. The majority of cardiovascular disorders ultimately culminate in heart failure, which impairs the heart’s capacity to pump and absorb blood as well as alters the composition and function of the heart’s muscle cells. It is crucial to remember that heart failure is the typical final stage of the pathological progression of CVD rather than an isolated illness. The majority of heart failure patients have left-sided heart failure at first, with pulmonary circulation congestion acting as the pathology’s initial symptom (Liu et al., 2021).

According to the research, Fan and colleagues demonstrated that exosomal circHIPK3 attenuates apoptosis in H2O2-treated AC16 cardiomyocytes, with exosomes isolated using the ultracentrifugation method and observed by transmission electron mi-croscopy. Functional studies revealed that this exosomal circRNA enhances AC16 cell proliferation and suppresses apoptosis under oxidative stress induced by H2O2. Mechanistically, circHIPK3 sequesters miR-33a-5p, leading to the upregulation of its target gene IRS1. Overexpression of miR-33a-5p counteracted the anti-apoptotic effect of exosomal circHIPK3 in H2O2-treated AC16 cardiomyocytes. Conversely, inhibition of miR-33a-5p promoted AC16 cell proliferation under oxidative stress, an effect that was nullified by IRS1 knockdown. These findings elucidate a novel pathological axis relevant to myocardial infarction (Fan et al., 2023) (Figure 3; Table 2).

Together with Ding et al., these studies reveal the functional versatility of circHIPK3 in cardiac protection. It operates not only as a scaffold protein regulator to combat senescence but also as a miRNA sponge to suppress apoptosis. Both mechanisms converge on enhancing cardiomyocyte survival and function, positioning exosomal circHIPK, particularly when delivered via UMSC-Exo as a multifaceted therapeutic candidate for a spectrum of cardiac conditions, from aging-related decline to acute ischemic injury.

### 4.5 Myocardial injury

Myocardial injury is the initial destructive event, which is caused by ischemia, infection, poisoning, and other reasons affecting myocardial cells, resulting in degeneration, necrosis, and a direct decrease in the number of myocardial cells, and the heart’s contractile and diastolic functions are impaired. Myocardial injury is the most common and direct inducement to initiate cardiac fibrosis.

Given the irreversible nature and severe consequences of myocardial injury, relevant studies have identified the mechanisms of action of exosomal circRNA, with the aim of developing new clinical diagnostic and therapeutic strategies. Tian et al. determined that exosomes from MSCs, which lack circ_0002113 (circIFNGR2), were found to attenuate myocardial infarction. This protective effect is mediated by sequestering miR-188–3p, which modulates runt-related transcription factor 1 (RUNX1) nuclear translocation, thereby reinforcing the molecular sponge mechanism of circRNAs. The alleviation of apoptosis through the circ_0002113/miR-188–3p/RUNX1 axis represents a novel therapeutic strategy for treating myocardial ischemia-reperfusion injury (Tian et al., 2022). Li et al. investigated the protective role of MSC-derived exosomes against sepsis-induced cardiac damage. FISH assay demonstrated the location of circRTN4 in the cytoplasm of cardiomyocytes. CircRTN4 could be delivered to cardiomyocytes via MSC-derived exosomes. The expression of circRTN4 was reduced in the cardiac tissues from caecal ligation and puncture rats and LPS-treated cardiomyocytes. The research elucidates a regulatory mechanism whereby MSC-exosomal circRTN4 modulates septic myocardial injury, positioning it as an anticipated therapeutic strategy for this condition (Li et al., 2022).

In addition, Lan et al. found that hypoxic cardiomyocytes release exosomes containing circSLC8A1. In the research, exosomes were isolated using ultracentrifugation and characterized through microscopic observation or protein detection. Protein expression was analyzed by Western blot. The expression levels of circSLC8A1, miRNA-214–5p, and TEA domain transcription factor 1 were quantified via qRT-PCR. Exosomal circSLC8A1 exacerbated the hypoxic effects by further suppressing cell viability while promoting inflammation, apoptosis, and oxidative stress. These findings indicate that circSLC8A1 exacerbates cellular injury in cardiomyocytes under hypoxic conditions by sponging miR-214–5p, thereby upregulating TEAD1 expression (Lan et al., 2022), providing clear directions for clinical diagnosis and drug development. Similarly, Zhou et al. demonstrated that exosomes from ADSCs, particularly the ones enriched with circ_0001747 (circChordc1), attenuated hypoxia/reoxygenation (H/R) -induced dysfunction in HL-1 cardiomyocytes. Mechanistically, overexpression of miR-199b-3p partially reversed the protective effects conferred by exosomal circ_0001747. Myeloid cell leukemia sequence 1 (MCL1) was identified as a direct target of miR-199b-3p. In HL-1 cells, silencing miR-199b-3p reduced H/R-induced damage, in part via upregulating MCL1. Circ_0001747 elevates MCL1 expression at both mRNA and protein levels by sequestering miR-199b-3p. Collectively, these findings indicate that ADSCs-derived exosomes rich in circ_0001747 can protect against H/R-induced HL-1 cell dysfunction (Zhou et al., 2022). This confirms that exosomal circRNAs play an indispensable role in myocardial injury repair, enriching the research content in this field.

Last but not least, Xie et al. found that exosomes use the circZNF292/miR-146a-5p axis to provide intrinsic regulatory signals to the heart. This discovery underscores the therapeutic potential for cardiovascular disorders linked to myocardial injury (Xie et al., 2024), further confirming the typical involvement of exosomal circRNAs in such pathological processes (Figure 3; Table 2). These research findings demonstrate that exosomal circRNA plays a highly active role in myocardial injury and holds significant therapeutic potential, making it a priority for further research.

### 4.6 Hypertrophic cardiomyopathy

Hypertrophic cardiomyopathy (HCM) is a primary disorder of the myocardium, defined by the presence of left ventricular hypertrophy without an identifiable cause, myofibrillar disarray, and interstitial fibrosis. It represents a critical pathological endpoint of maladaptive cardiac remodeling.

High levels of circ_0018553 in endothelial progenitor cell-derived exosomes were found by Zuo et al. RNA expression analysis showed that circ_0018553 acts as a molecular sponge for miR-4731 in an *in vitro* study of angiotensin II (Ang II)-induced cardiac hypertrophy. By attaching to the 3′untranslated region of sirtuin 2 (SIRT2), this miRNA suppresses its production. Overexpression of miR-4731 exacerbated cardiac hypertrophy in the Ang II-treated model. Conversely, SIRT2 silencing produced the opposite effect. The study shows that by modulating the miR-4731/SIRT2 signaling pathway, exosomal circ_0018553 reduces cardiac hypertrophy (Zuo et al., 2023) (Figure 3; Table 2).

This study is confined to *in vitro* experiments, therefore, the mechanistic function of circ_0018553 in cardiac hypertrophy requires further validation in animal models *in vivo*. Additionally, the hypertrophy induced by Ang II or hypertension progresses through multiple stages, beginning as adaptive hypertrophy and advancing to maladaptive remodeling, which involves further myocyte hypertrophy, heightened apoptosis, and remodeling of the extracellular matrix. Because of the complex mechanism of cardiac hypertrophy, there is a lack of effective treatments clinically. Accumulating evidence indicates that circRNAs play a regulatory role in cardiac hypertrophy. This underscores the rationale for exploring novel therapeutic strategies and potential pharmacological interventions targeting circRNAs.

### 4.7 Dilated cardiomyopathy

Dilated cardiomyopathy (DCM) is a genetic primary myocardial disease characterized by impaired contractile function of the left or both ventricles. The onset of the disease is related to viral infection and genetic factors. Clinical manifestations include dyspnea, edema, weakness, and respiratory. Most patients with this disease cannot be cured. Drug therapy is mainly used for treatment. The prognosis is poor. After drug therapy, the condition can be maintained stable. If treated in time, patients may develop severe conditions such as heart failure. Clinical issues such as incurability and poor prognosis urgently need to be addressed.

Xu et al. identified 49 circRNAs specifically expressed in the DCM with chronic heart failure cohort. These circRNAs were linked to several critical biological processes, including extracellular ligand-receptor segregation, N-acetyltransferase and histone acetyltransferase activities, and endocytic vesicle membrane composition. The findings enhance our understanding of DCM pathophysiology and support the refinement of clinical diagnostic approaches (Xu et al., 2024) (Figure 3; Table 2).

### 4.8 Endothelial dysfunction

Endothelial dysfunction is defined as the impaired ability of the arterial endothelium to maintain its normal physiological functions. When endothelial dysfunction occurs, due to damage or abnormal function of endothelial cells, the normal regulation of VSMC contraction and relaxation cannot be maintained, resulting in increased sensitivity of the cerebral blood vessels to various stimuli such as catecholamines and 5-hydroxytryptamine. When the concentration of these substances increases, it can cause contraction of the cerebral blood vessels, reducing local blood flow.

According to Liu et al., through bioinformatic analysis of the host genes of differentially expressed circRNAs, enrichment was observed in vascular diseases and in several pathways associated with vascular pathology, such as focal adhesion, tight junction, and adherens junction. Subsequently, a ceRNA network was constructed. The differentially expressed mRNAs within this ceRNA network were then subjected to functional enrichment analysis using Ingenuity Pathway Analysis. The results of their research indicate that hsa_circ_0012627, hsa_circ_0053261, and hsa_circ_0052810 are linked to vascular endothelial dysfunction (Liu Q. et al., 2024).

Separately, Liu and colleagues reported that treatment with oxidized low-density lipoprotein (Ox-LDL) induced dysfunction in human umbilical vein endothelial cells (HUVECs). Expression of circ_0026218 (circCERS5) was elevated in AS serum samples and in HUVECs exposed to ox-LDL. Moreover, exosomes derived from ox-LDL-treated HUVECs showed increased levels of circ_0026218, indicating that it can be transferred via exosomes. Silencing circ_0026218 alleviated the ox-LDL-induced dysfunction in HUVECs. Circ_0026218 alleviates oxidized low-density lipoprotein-induced endothelial dysfunction by modulating the miR-188–3p/TLR4/NF-κB signaling pathway (Liu et al., 2024b) (Figure 3; Table 2). In addition, Zhang et al. demonstrated that circ_0004104 downregulation reduced ox-LDL-induced injury in human umbilical vein endothelial cells through miR-942–5p and rho-associated coiled-coil containing protein kinase 2 (ROCK2) (Zhang Y. et al., 2022). These findings collectively suggest that circRNAs function as molecular decoys, thereby modulating downstream signaling pathways involved in endothelial dysfunction. The identification of these specific circRNAs provides potential biomarkers for diagnosing vascular diseases and novel therapeutic targets to preserve endothelial integrity.

### 4.9 Other cardiovascular diseases

Exosomal circRNAs are likely implicated in the pathogenesis or progression of additional cardiovascular disorders. A growing body of research has directly or indirectly elucidated their specific functions in various cardiovascular conditions, highlighting their broad application potential in this field. For instance, Guo et al. demonstrated that circ_0036176 (circMYO9A), which is formed through the back-splicing of exon 2 to exon 4 of the myosin IXA gene, was found to be elevated in the myocardium of heart failure patients. Additionally, it was enriched in exosomes derived from human AC16 cardiomyocytes that overexpressed circ_0036176. It suppresses cardiac fibroblast proliferation via translation of the Myo9a-208 protein, which subsequently inhibits the cyclin/Rb pathway, which could be a promising target for treating myocardial fibrosis (Guo et al., 2022).

Moreover, detected by qRT-PCR and western blotting, circ_0001312 (circCCDC66) was demonstrated to counteract doxorubicin-induced cytotoxicity in cardiomyocytes through the miR-409–3p/HMGB1 (high mobility group box 1) axis. Mechanistically, circ_0001312 competitively binds to miR-409–3p, resulting in the upregulation of HMGB1, a direct target of miR-409–3p. In cardiomyocytes, doxorubicin (DOX) treatment reduced miR-409–3p levels while increasing HMGB1 expression. This circRNA is also secreted into the extracellular environment via exosomes (Hu et al., 2023). The study by Zhang et al., comparing high-throughput sequencing of serum exosomes obtained from three patients with venous malformation (VM) and three healthy donors, was performed to identify differentially expressed (DE) circRNAs, lncRNAs, and mRNAs implicated in the development of VM. The research found that delineated networks of differentially expressed ncRNAs within exosomes are associated with VM pathogenesis. In spite of limited data, this analysis offers novel perspectives on potential therapeutic targets for VM (Zhang Y. et al., 2023). Finally, in Han’s study, exosomes were isolated from three patients with type 2 diabetes mellitus (T2DM) and three T2DM patients with lower limb vascular disease (LLVD), and the circRNAs extracted from these exosomes were subjected to microarray analysis. Subsequently, five candidate biomarkers identified from the differentially expressed circRNAs were validated using quantitative real-time polymerase chain reaction in a larger cohort consisting of 20 T2DM patients and 20 T2DM patients with LLVD. The results show that hsa_circ_0001842 was identified as a potential biomarker for lower extremity vascular disease in individuals with type 2 diabetes mellitus (Liu et al., 2023) (Figure 3; Table 2).

## 5 Therapeutic potential of exosomal circRNAs in cardiovascular diseases

### 5.1 Potential therapeutic targets

Exosomal circRNAs impact the pathological progression of CVDs by regulating key signaling, making them highly attractive therapeutic intervention targets. This review summarizes various mechanisms of study in which specific exosomal circRNAs are targeted to ameliorate disease phenotypes.

Specific circRNAs were found to exert protective effects by inhibiting cell death pathways. For example, the delivery of circ_0001747, which targets the miR-199b-3p/MCL1 axis, can suppress H/R-induced cardiomyocyte apoptosis (Zhou et al., 2022), while circ_0001312 reversed the cytotoxic effects mediated by DOX on cardiomyocytes (Hu et al., 2023). These studies indicate that intervening in these well-defined circRNA mechanisms can precisely regulate downstream cell death and inflammatory pathways.

Secondly, circRNAs possess unique functions in myocardial repair and regeneration. CircASXL1 plays an active part in cardiac repair following myocardial infarction by synergistically promoting cardiomyocyte cell cycle re-entry and cytokinesis, offering a new strategy for cardiac regeneration (Wang Y. et al., 2024). This suggests that circRNAs can not only suppress damage but also actively promote repair.

Furthermore, circRNAs participate in regulating age-related functional decline. For example, circHIPK3 efficiently reduces p21 activity by encouraging the ubiquitination and degradation of HuR, which delays cardiomyocyte senescence and cardiac failure. This indicates that the regulatory network of circRNAs also extends to aging-related pathways (Ding et al., 2022).

In summary, circRNAs in exosomes constitute a multi-layered, interconnected myocardial protection network through various mechanisms, including the inhibition of cell death, promotion of tissue repair, and delay of cellular senescence. Interventions targeting these well-defined circRNAs and their downstream pathways can provide clear molecular targets and a logical framework for the development of precise, targeted therapeutic agents.

### 5.2 Diagnostic biomarkers

Due to their characteristics of high stability, tissue/disease-specific expression, and easy access from body fluids such as blood in a non-invasive manner, exosomal circRNAs demonstrate significant potential for the early detection and prognostic assessment of CVDs. A considerable proportion of the studies have focused on discovering and validating exosomal circRNAs as disease biomarkers. For example, it has been demonstrated that the combination of differentially produced circRNAs, such as hsa_circ_000160 and hsa_circ_0000038, found in the plasma exosomes of individuals with coronary artery disease, may be useful as diagnostic biomarkers (Liu X. et al., 2023; Tong et al., 2023b; Zhang et al., 2024b). In patients with acute myocardial infarction, hsa_circ_001558 and exo_circ_0020887 have also been considered as potential prognostic and diagnostic indicators (Wang et al., 2023). These studies lay the foundation for developing non-invasive liquid biopsy technologies based on exosomal circRNAs.

### 5.3 Exosomes as new carriers of therapeutics and drugs

Exosomes possess inherent advantages, such as high biocompatibility, low immunogenicity, and the capacity to traverse biological barriers, positioning them as ideal candidates for drug delivery systems. By loading exosomal RNAs with therapeutic effects or molecules capable of regulating endogenous circRNAs into engineered exosomes, targeted delivery to detailed diseased cells is promising to be achieved. For example, exosomes derived from human UMSC-Exo are effective carriers for delivering functional circRNAs such as circHIPK3 and circASXL1, successfully improving phenotypes such as cardiac aging and myocardial injury in animal models (Ding et al., 2022; Wang Y. et al., 2024). This provides proof-of-concept for the development of exosome-based “cell-free gene therapies for CVDs. By engineering modifications to the exosome membrane to enhance their homing ability to cardiac or vascular diseased sites, their therapeutic specificity and efficacy will be further improved in the future.

### 5.4 Traditional Chinese medicine-mediated regulation of exosomal circRNAs in cardiovascular diseases

Compared with single-target chemical interventions, accumulating evidence has clearly validated the reliable role of traditional medicine in regulating circRNAs and exosomal circRNAs, as well as in intervening in CVDs (Zhu et al., 2024). Compared with other regulatory approaches, bioactive substances derived from traditional medicine possess unique advantages of high efficiency and mildness in modulating exosomal circRNAs and ameliorating the progression of CVDs (Li X. H. et al., 2022; Liu Y. et al., 2024). Among these, traditional warming-yang drugs and formulae (e.g., Sini Decoction, composed of Aconiti Radix Lateralis Praeparata, Zingiberis Rhizoma, and Glycyrrhizae Radix et Rhizoma) can effectively alleviate tumor therapy-related cardiotoxicity, which also affects the regulation of exosomal circRNAs (Zhang et al., 2021). Specifically, extracts of Aconiti Radix Lateralis Praeparata and Zingiberis Rhizoma can upregulate the expression of exosomal circ_Ttc3, which targets and activates the Arl2 pathway by sponging miR-15b, thereby reducing cardiomyocyte apoptosis. Circ_Foxo3 is highly expressed in exosomes derived from serum containing Sini Decoction, which can target and inhibit miR-136 while activating the SIRT1 antioxidant axis to protect myocardial mitochondrial function. Additionally, total alkaloids from Aconiti Radix Lateralis Praeparata can promote the release of exosomal circ_ANRIL, thereby reversing chemotherapy-induced myocardial fibrosis (Zhang et al., 2024a). In contrast to single-target chemical interventions, the regulation of exosomal circRNAs by traditional medicine is more holistic and gentle, offering a novel direction for the inhibition and treatment of tumor therapy-related cardiotoxicity as well as for research on exosomal circRNA regulation (Xu et al., 2025).

## 6 Conclusions and perspectives

Exosomal circRNAs play a significant mechanistic role in diverse CVDs, including CHD, AS, cellular senescence, myocardial infarction, myocardial injury, cardiac hypertrophy, DCM, endothelial dysfunction, and heart failure, in which the molecular sponge mechanism is the most elucidated so far, together with protein scaffold, protein recruiter, peptide translation, and RNA interaction, achieving precise regulation of biological effects in CVDs.

However, quantitative analytical data remain limited. The accurate identification and quantification of exosomal circRNAs remain technically demanding. Meanwhile, the relevant theories failed to form a complete system. A subset of studies established correlations between the content change of exosomal circRNA and the disease, lacking further experimental research on the mechanism. Moreover, no typical clinical trials related to the topic have been found at present. Further research is needed and clinical application remains a distant goal.

In conclusion, exosomal circRNAs constitute critical players in the pathogenesis, diagnosis, and therapy of CVDs. Their capacity to act as miRNA sponges underpins a wide range of regulatory effects, and emerging evidence from omics studies highlights their potential as biomarkers for refining diagnostic standards and identifying therapeutic targets. Upon additional experimental investigation, these molecules are expected to translate into broad clinical utility.
